# Association between skeletal muscle mitochondrial dysfunction and insulin resistance in patients with rheumatoid arthritis: a case–control study

**DOI:** 10.1186/s13075-023-03065-z

**Published:** 2023-05-20

**Authors:** Douglas R. Moellering, Kelley Smith-Johnston, Christian Kelley, Melissa J. Sammy, Jason Benedict, Guy Brock, Jillian Johnson, Kedryn K. Baskin, Wael N. Jarjour, Martha A. Belury, Peter J. Reiser, Prabhakara R. Nagareddy, Beatriz Y. Hanaoka

**Affiliations:** 1 Department of Nutrition Sciences, School of Health Professions, University of Alabama at Birmingham, Birmingham, AL USA; 2 Center for Exercise Medicine, University of Alabama at Birmingham, Birmingham, AL USA; 3 Department of Biomedical Bioinformatics, College of Medicine, The Ohio State University, Columbus, OH USA; 4 Department of Surgery, College of Medicine, The Ohio State University, Columbus, OH USA; 5 Department of Physiology and Cell Biology, College of Medicine, The Ohio State University, Columbus, OH USA; 6 Division of Rheumatology and Immunology, Department of Internal Medicine, The Ohio State University, Columbus, OH USA; 7 Department of Human Sciences, College of Education and Human Ecology, The Ohio State University, Columbus, OH USA; 8 Division of Biosciences, College of Dentistry, The Ohio State University, Columbus, OH USA

**Keywords:** Rheumatoid arthritis, Skeletal muscle, Mitochondria

## Abstract

**Background:**

Insulin resistance affects a substantial proportion of patients with rheumatoid arthritis (RA). Skeletal muscle mitochondrial dysfunction results in the accumulation of lipid intermediates that interfere with insulin signaling. We therefore sought to determine if lower oxidative phosphorylation and muscle mitochondrial content are associated with insulin resistance in patients with RA.

**Methods:**

This was a cross-sectional prospective study of RA patients. Matsuda index from the glucose tolerance test was used to estimate insulin sensitivity. Mitochondrial content was measured by citrate synthase (CS) activity in snap-frozen muscle samples. Mitochondrial function was measured by using high-resolution respirometry of permeabilized muscle fibers and electron transport chain complex IV enzyme kinetics in isolated mitochondrial subpopulations.

**Results:**

RA participants demonstrated lower insulin sensitivity as measured by the Matsuda index compared to controls [median 3.95 IQR (2.33, 5.64) vs. 7.17 (5.83, 7.75), *p* = 0.02]. There was lower muscle mitochondrial content among RA vs. controls [median 60 mU/mg IQR (45, 80) vs. 79 mU/mg (65, 97), *p* = 0.03]. Notably, OxPhos normalized to mitochondrial content was higher among RA vs. controls [mean difference (95% CI) = 0.14 (0.02, 0.26), *p* = 0.03], indicating a possible compensatory mechanism for lower mitochondrial content or lipid overload. Among RA participants, the activity of muscle CS activity was not correlated with the Matsuda index (*ρ* =  − 0.05, *p* = 0.84), but it was positively correlated with self-reported (IPAQ) total MET-minutes/week (*ρ* = 0.44, *p* = 0.03) and Actigraph-measured time on physical activity (MET rate) (*ρ* = 0.47, *p* = 0.03).

**Conclusions:**

Mitochondrial content and function were not associated with insulin sensitivity among participants with RA. However, our study demonstrates a significant association between muscle mitochondrial content and physical activity level, highlighting the potential for future exercise interventions that enhance mitochondrial efficiency in RA patients.

**Supplementary Information:**

The online version contains supplementary material available at 10.1186/s13075-023-03065-z.

## Background

Rheumatoid arthritis (RA) is a systemic autoimmune disease associated with a high prevalence of insulin resistance [1, 2], a known risk factor for cardiovascular disease and type 2 diabetes [3]. Several studies have reported that insulin resistance in RA is associated with systemic inflammation, rheumatoid factor and anti-citrullinated protein antibodies (ACPA) seropositivity, obesity, and glucocorticoid use [4–6]. Although the precise mechanisms of insulin resistance have not been reported, impaired muscle mitochondrial metabolism could be involved. Currently, literature indicate that increased fatty acid uptake into muscle due to lipid overload or impaired oxidation of fatty acids by muscle mitochondria results in intramyocellular accumulation of toxic lipid species (e.g., ceramides and diacylglycerols) that interfere with insulin signaling and contribute to insulin resistance [7, 8].

The aim of this study was to determine if lower oxidative phosphorylation (OxPhos) and muscle mitochondrial content are associated with insulin resistance in RA. In sedentary individuals with insulin resistance or type 2 diabetes, there are several reports that muscle mitochondria are smaller, less abundant, or exhibit lower oxidative capacity compared to controls [9–13], although some studies found no differences between these groups [14, 15]. In RA, there is limited information about muscle mitochondria, and the data that exist is focused on the effects of inflammation on skeletal muscle mass and contractile function [16–19]. We undertook investigation of mitochondrial function to precisely measure alterations of cellular energy metabolism in RA, in order to better assess future therapeutic interventions for improving outcomes in RA patients, including exercise and diet [20].

In the present study, CS activity and complex IV activity were evaluated as complimentary assessments of mitochondrial content and function, respectively.

## Participants and methods

### Participants

This prospective, cross-sectional study was conducted at the University of Alabama Birmingham, in Birmingham, AL, and at the Ohio State University, in Columbus, OH, between March of 2017 and January of 2022. This study was approved by both institutional review boards and has therefore been performed in accordance with the ethical standards laid down in the 1964 Declaration of Helsinki and its later amendments. All participants provided their written informed consent to participate. Participants between 35 and 65 years who met the 2010 ACR /EULAR criteria for RA [21] and age/sex/body mass index (BMI) matched healthy individuals were enrolled as controls. Participants with type 2 diabetes mellitus or a history of resistance training were excluded. More detailed participant eligibility criteria have been previously reported [22]. Race and ethnicity were assessed by self-report from a fixed set of categories. Data on RA disease duration, use of disease-modifying treatments for RA (disease-modifying antirheumatic drugs [DMARDs] and biologic agents) and glucocorticoids (daily dose of prednisone); co-morbidities and smoking status were obtained from the study participants and medical records.

### Clinical tests

#### Anthropometric measures

Height and weight were measured using a stadiometer (SECA 216 Accu-Hite Measuring Device, Seca North America, Chino CA) and an electronic digital scale (SECA 1360 Wireless scale, Seca North America, Chino CA), respectively. BMI was calculated using the usual formula (weight [kg]/height [m]^2^). Waist and hip circumferences were measured using a tape with the participant in a standing position. Waist circumference was measured at the midpoint between the lower rib and the upper margin of the iliac crest. Hip circumference was assessed at the widest circumference around the buttocks, below the iliac crest. Both measurements were taken twice by the same individual, using the same tape, and were recorded to the nearest 0.1 cm. Those whose two waist or hip measurements differed by more than 3 cm had a third measurement taken. The mean of the two valid measurements was used in our analysis.

#### Disease activity of RA

The Disease Activity Score 28-joint count C-reactive protein (DAS28-CRP) score was calculated. This score is based on clinical assessment of tenderness and swelling in 28 joints, the serum level of high-sensitivity C-reactive protein (hsCRP), and a general health assessment on a visual analog scale [23].

#### Self-reported physical activity

Participants were asked to complete the IPAQ-Long Form. The IPAQ questionnaire records the last 7-day recall for four intensity levels of physical activity which is vigorous-intensity activity, moderate-intensity activity, walking, and sitting. From IPAQ, data were converted to Metabolic Equivalent minutes per week (MET-min/week) using the published formulation [24].

#### Accelerometer

Participants were instructed to wear a commercially available accelerometer (model wGT3X-BT, ActiGraph, Pensacola, FL) on their hip during waking hours for seven consecutive days, except during conditions that could potentially damage the device (e.g., water-based activities). Participants meeting valid wear time requirements (≥ 4 days of ≥ 8 h/day) were included in the analysis. All data were acquired in 1-min epochs. The software ActiLife v6.13.4 (Actigraph, Pensacola, FL) was used to analyze the actigraphy data and obtain step counts/day, metabolic equivalents (METs), total activity count/day and moderate-to-vigorous physical activity/day (MVPA/day).

#### DXA scans

The total body fat mass and fat-free mass were measured by dual-energy X-ray absorptiometry (DXA) using a Lunar Prodigy densitometer (GE-Lunar Radiation Corp. Madison, WI). Fat-free mass index (FFMI) was calculated as fat-free body mass (kg) /height (m^2^). Fat mass index was calculated as total body fat mass /height (kg/m^2^).

#### Oral Glucose Tolerance Test (OGTT)

After an overnight fast of at least 8 h, participants underwent the 75 g 2-h OGTT test. Blood samples were drawn at 0, 30, 60, 90, and 120 min. Insulin sensitivity was calculated using the OGTT data according to Matsuda and DeFronzo (Matsuda index) [25]. Although insulin resistance usually develops simultaneously in multiple organs, the severity of insulin resistance can vary among different tissues. The Matsuda index has been shown to be strongly correlated with the rate of whole-body (mainly skeletal muscle) glucose disposal in euglycemic insulin clamp studies [26].

#### Serum biochemical measures

Serum glucose assays were performed on an automated glucose analyzer (Sirrus analyzer; Stanbio Laboratory, Boerne, TX), and serum insulin was measured using an immunofluorescent method with a 900 AIA analyzer (TOSOH Bioscience, South San Francisco, CA). Insulin sensitivity was estimated using the Matsuda index, which has been shown to correlate well with whole-body insulin sensitivity measured by the euglycemic insulin clamp technique [25]. Serum level of hsCRP was measured by immunoturbidimetry (Beckman Coulter, Brea, CA, USA).

### Tissue collection and laboratory analyses

#### Needle biopsy of the vastus lateralis muscle

Muscle samples were collected from the vastus lateralis. Briefly, biopsies were performed under aseptic conditions and local anesthetic (1% lidocaine) using a 5-mm Bergstrom-type biopsy needle under suction, as previously described [27].

#### Preparation of muscle biopsies

The muscle biopsy (approximately 200 mg) was divided into three parts. One part was prepared for respiratory measurements in saponin-permeabilized muscle fibers (PMF), another part was mounted on cork using gum tragacanth and frozen in liquid nitrogen-cooled isopentane for histology, and a third part was snap frozen in liquid nitrogen and stored at − 80 °C until further analysis of enzyme activities and myosin heavy chain isoform quantification.

#### Muscle histology

Human skeletal muscle is composed of three major fiber types referred to as slow type I and fast IIA and fast IIX fibers. Fiber types I and IIA have greater oxidative capacity compared to type IIX fibers [28], and therefore CS activity and mitochondrial content are fiber type dependent. As such, we quantified muscle type distribution by myosin heavy chain isoform immunohistochemistry in a subset of RA and control participants(16 RA and 16 controls), using established methods, as previously described [29, 30].

#### Myosin heavy chain (MHC) isoforms

A portion of each biopsy (15 controls and 14 RA) was prepared for analysis with gel electrophoresis, as described previously [31]. The relative amounts of fast-type MHC-IID/X and MHC-IIA and of slow-type MHC-I were determined using ImageJ software. Based upon the dimensions (width, depth, and length) of human single fibers that were studied for a separate project (not published) and the mass and dimensions of the biopsy samples, we estimate that there were approximately 1,300 fibers in each homogenate. Therefore, the gel electrophoresis results were based upon analysis of a much larger number of fibers than what is practical to analyze using the immunohistochemical approach.

##### Muscle mitochondrial preparations

The subsarcolemmal (SS) and intermyofibrillar (IMF) fractions of skeletal muscle mitochondria were isolated using our custom-built homogenizer as previously described [32].

##### Ex vivo mitochondrial studies

Methods to study mitochondrial function ex vivo include evaluation of individual respiratory chain complexes’ kinetic activity and high-resolution respirometry of isolated mitochondria or permeabilized muscle fibers. Respirometry is currently the “gold standard” measurement of mitochondrial oxidative phosphorylation evaluating the coupled production of ATP to the flow of electrons through the electron transport chain (ETC) culminating in the measurement of the reduction of oxygen. This flow of electrons through the ETC occurs through transfer of electrons through a chain of multi-protein complexes (complexes I-IV) which generate an electro-chemical gradient that acts as the driving force for ATP synthesis.

Phenotypic analysis of the skeletal muscle individual mitochondrial respiratory chain complexes’ kinetic activity is widely employed to measure mitochondrial function, since frozen samples may be used [33]. CS activity is a surrogate biomarker of skeletal muscle mitochondrial volume and is strongly correlated with the gold standard method for mitochondrial quantification in tissues, e.g. transmission electron microscopy [34]. Complex IV activity is closely associated with muscle OxPhos, as determined by the maximal couple respiration rate (state 3) in permeabilized fibers [34].

#### Citrate synthase (CS) activity

CS activity was measured (24 RA and 20 controls) using the coupled reaction with oxaloacetate, acetyl-CoA, and 5,5-dithiobis-(2,4-nitrobenzoic acid), as previously described [35].

#### Electron transport chain complex IV activity

Complex IV activity was measured (18 RA and 8 controls) by the oxidation of cytochrome c at 550 nm as previously described. Enzyme activity was determined at 37 °C on a Beckman Coulter DU 800 Spectrophotometer. Data are represented as the pseudo first order rate constant (k) divided by protein concentration. Measurement of ETC complex IV activity in isolated mitochondria was performed to corroborate the respirometry results in permeabilized muscle fibers, as well as to assess the subsarcolemmal and intermyofibrillar mitochondria subpopulations. These are two distinct subpopulations of mitochondria in skeletal muscle that exhibit unique biochemical and functional properties [36].

#### Mitochondrial respiratory protocol

Skeletal muscle samples were collected and permeabilized (19 RA and 9 controls), as previously described [37, 38]. Briefly, samples were immediately transferred to ice-cold transport buffer (10 mM EGTA, 0.1uM free calcium, 20 mM imidazole, 20 mM taurine, 50 mM K-MES, 6.56 mM MgCl_2_, 5.77 mM ATP, 15 mM phosphocreatine). Samples were treated with 30 µg/mL saponin to permeabilize the fibers and gently rotated for 30 min at 4 °C (Thermo Scientific Tube Rocker). The samples were then washed 1 × in MiR03 buffer (containing in mM – 0.5 EGTA, 3 MgCl2.6H2O, 20 Taurine, 10 KH2PO4, 20 HEPES, 200 Sucrose, 1 g/L BSA) by gentle rotation for 15 min at 4 °C and then transferred to fresh MiR03 buffer for high-resolution respirometry (HRR).

HRR was performed by measuring oxygen consumption in 2 mL of MiR03- creatine/blebbistatin buffer, in a two-chamber respirometer (Oroboros Oxygraph-2 k with DatLab software; Oroboros Instruments Corp., Innsbruck Austria) with constant stirring at 750 rpm. Respiration rates were measured using malate (5 mM) and palmitoylcarnitine (40uM) to determine the leak rate with the fatty acid substrate, palmitoylcarnitine. Next, 5 mM ADP was added to determine the OXPHOS capacity at saturating ADP concentrations. Ten micromolar Cytochrome C was added as a measure of membrane integrity (a response > 10% over state 3 was considered damaged and excluded from the dataset). Maximal rate of uncoupler-stimulated respiration was measured using increasing doses of 2-[2-[4-(trifluoromethoxy) phenyl] hydrazinylidene]-propanedinitrile (FCCP) from 0.5 to 1.25 µM. Electron flow through complex IV was then measured after inhibition of complex III by antimycin A and by adding Ascorbate (As, 2 mM) and tetramethyl-p-phenylenediamine (TMPD, 0.5 mM). Correction for the autoxidation of TMPD/As was performed. TMPD is a Complex IV-specific electron donor and ascorbate ensures TMPD is reduced for a continuous flow of electrons to generate a linear rate of Complex IV activity, which also approximates mitochondrial mass or volume and was used to bioenergetically normalize all respirometry data. The respiratory acceptor control ratio (RCR 3/2) was calculated as the ratio of OxPhos respiration (state 3) to LEAK respiration (state 2) as an indicator of the mitochondrial coupling state [39].

### Statistical analysis

Distributions of all variables were evaluated using bar charts for categorical variables and histograms for continuous variables. Descriptive statistics were presented as frequencies and percentages (%) for categorical variables, and mean (standard deviation) or median (interquartile range, IQR) for continuous variables, depending on the normality of the variable. Two-sample *t*-tests (for normally distributed variables) or Man-Whitney *U* tests (for non-normally distributed variables) tested differences in continuous outcomes between RA and control groups. Chi-squared tests assessed the association between categorical variables with Fisher’s exact test for tables with small counts. Spearman’s correlations evaluated the association between continuous variables of interest. To account for multiple chambers per individual, mixed effects models with chamber as a fixed effect and individual as a random effect tested differences in mitochondrial function between RA and control groups, for both weight- and mitochondrial-content standardized respirometry data.

All statistical analyses were performed using R version 4.1.2. *P* values < 0.05 were considered to be statistically significant.

## Results

### Characteristics of study participants

A total of 24 RA participants and 20 non-RA controls were enrolled in this study. The median (IQR) age of RA participants was 57 (50, 61) years. The RA participants were predominantly female (88%) and white (88%) and overweight, with a median BMI (IQR) of 28.5 (24.7, 30.1) kg/m^2^. Systemic hypertension was more frequent among RA participants (25%) compared to controls (0%), *p* = 0.03. Dyslipidemia was also more frequent among RA participants (29%) compared to controls (5%), *p* = 0.05). There were no significant differences between RA participants and non-RA controls in terms of age, sex, race or BMI, smoking status, waist-to-hip ratio, lean mass (FFMI), fat mass (FMI), or serum levels of hsCRP. General characteristics, anthropometric measures, DXA body composition, physical performance tests, and biochemical parameters of the total study population, comprising of RA participants and non-RA controls, are presented in Table 1.

**Table 1:** Characteristics of study participants (total study population)

Characteristic	Control (*n* = 20)	RA (*n* = 24)	*p*-value
Age, years	56 (46, 60)	57 (50, 61)	0.77
Female sex (%)	17 (85)	21 (88)	> 0.99
Race (%)			0.81
White	17 (85)	21 (88)	
Black or African American	3 (15)	2 (8)	
More than one race	0 (0)	1 (4)	
Smoking status (%)			0.26
Never smoked	16 (80)	13 (54)	
Past smoker	2 (10)	6 (25)	
Current smoker	2 (10)	5 (21)	
Co-morbidities			
Dyslipidemia (%)	1 (5)	7 (29)	0.05
Systemic hypertension (%)	0 (0)	6 (25)	0.03
COPD/emphysema (%)	0 (0)	1 (4)	> 0.99
Anthropometrics			
BMI, kg/m^2^	26.1 (23.3, 30.8)	28.5 (24.7, 30.1)	0.36
Waist-to-hip ratio	0.85 (0.82, 0.90)	0.89 (0.85, 0.93)	0.10
Fat-free mass index (kg/m^2^)	15.6 (14.9, 17.3)	16.4 (15.5, 17.6)	0.51
Fat mass index (kg/m^2^)	9.8 (7.2, 12.0)	11.4 (8.8, 13.2)	0.31
Physical activity measures			
IPAQ, total MET-minutes/week	4810 (2210, 13,150)	1598 (1043, 4984)	< 0.01
IPAQ Moderate intensity MET-min/week	2055 (120, 4,178)	1155 (409, 2632)	0.35
IPAQ Vigorous intensity MET-min/week	1080 (0, 3,420)	0 (0, 0)	< 0.01
Accelerometer (*n* = 19, 22)			
MET Rate (kcals hourly equivalent)	1.11 (1.07, 1.19)	1.05 (1.02, 1.07)	< 0.01
Total activity count/day (in millions)	1.41 (0.88, 1.83)	0.93 (0.73, 1.16)	0.06
MVPA/day	121 (87, 149)	94 (43, 114)	0.03
Step counts/day	10,499 (8354, 13,429)	5394 (3721, 8748)	< 0.01
Serum C-reactive protein mg/L (*n* = 20,21)	1.34 (0.75, 2.69)	1.11 (0.54, 1.55)	0.33
Matsuda index	7.17 (5.83, 7.75)	3.95 (2.33, 5.64)	0.02

Table S1 presents the characteristics of the subgroup of RA and non-RA controls with mitochondrial respirometry data, which were largely comparable to the total study population in Table 1.

Our data show that RA participants demonstrated significantly lower levels of insulin sensitivity. The median Matsuda index (IQR) was 3.95 (2.33, 5.64) among RA participants compared to 7.17 (5.83, 7.75) among controls (*p* = 0.02). RA participants also reported significantly lower levels of physical activity. The median IPAQ total MET-minutes per week (IQR) was 1598 (1043, 4984) among RA participants compared to 4810 (2210, 13,150) among controls (*p* < 0.01); the median IPAQ vigorous intensity MET-minutes per week (IQR) was 0 (0,0) among RA participants compared to 1080 (0, 3420) among controls (*p* < 0.01), the median step counts per day (IQR) was 5,394 (3721, 8748) among RA participants compared to 10,499 (8354, 13,429) among controls (*p* < 0.01), and the median MVPA/day was 94 (43, 114) among RA participants compared to 121 (87, 149) among controls (*p* = 0.03).

### Clinical characteristics of participants with RA

The clinical characteristics of participants with RA are shown in Table 2. Approximately two thirds of RA participants were positive for either rheumatoid factor (RF) or anti-cyclic citrullinated peptide (anti-CCP). The median disease duration (IQR) was 7 (3, 13) years. The median DAS-28 CRP (IQR) was 1.77 (1.23, 3.13), which corresponds to low disease activity. All participants were on DMARD therapy for RA. Only 13% of RA participants were on prednisone and the mean prednisone dose (SD) was 2 (8) mg/day.

**Table 2:** Clinical characteristics of participants with RA

Characteristic	RA (*n* = 24)
RF positive, *n* (%)	14 (63)
Anti-CCP positive, *n* (%)	14 (58)
RF or anti-CCP positive, *n* (%)	16 (67)
Disease duration (years), median (IQR)	7 (3, 13)
DAS-28 CRP, median (IQR) *n* = 23	1.77 (1.23, 3.13)
Prednisone use, *n* (%)	
Current	3 (13)
Past	19 (79)
Never taken	2 (8)
Current prednisone dose (mg/day), median (IQR)	5 (5, 5)
Patients on any DMARD, *n* (%)	24 (100)
Current DMARD use, *n* (%)^a^	
Methotrexate	8 (33)
Hydroxychloroquine	7 (29)
Sulfasalazine	0 (0)
Etanercept	2 (8)
Adalimumab	6 (25)
Infliximab	1 (4)
Abatacept	0 (0)
Rituximab	1 (4)
Tocilizumab	2 (8)
Tofacitinib	3 (12)

^a^Patients could be taking more than one DMARD at a time

### Muscle mitochondrial function

**Fig. 1:**
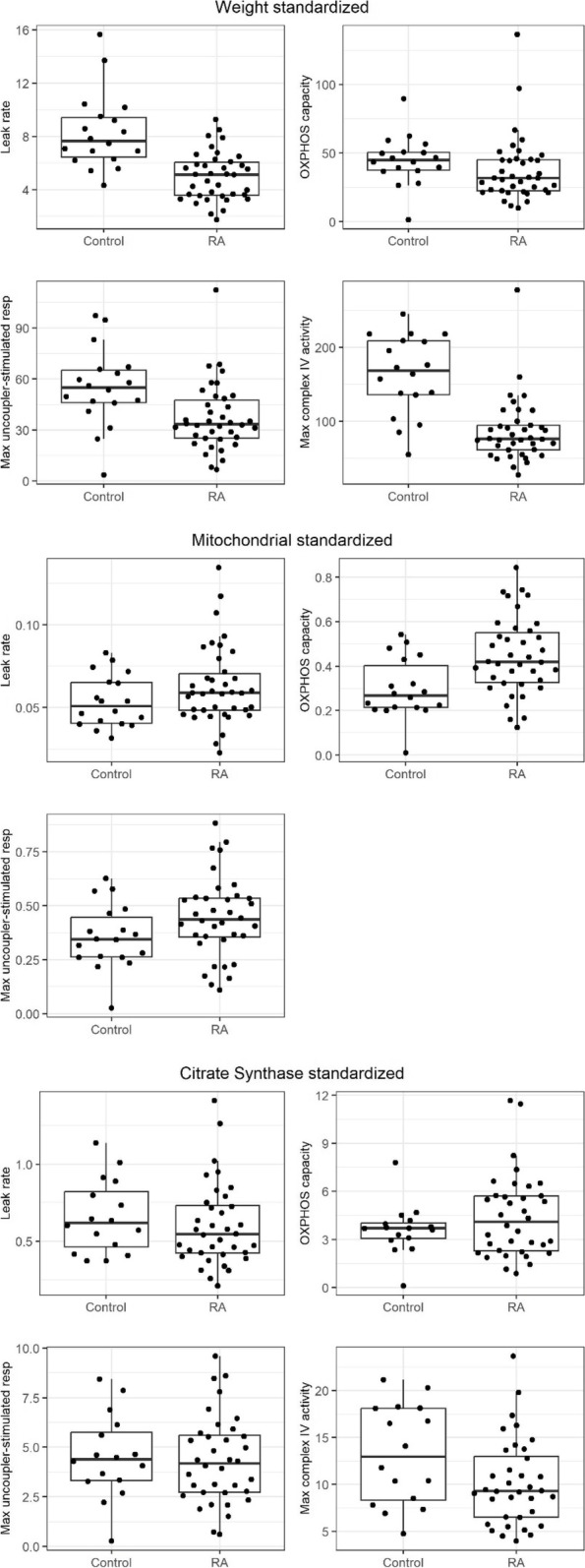
High-resolution respirometry data of RA and control permeabilized skeletal muscle fibers. Median leak rate with fatty acid substrate (PC), OxPhos capacity and maximal rate of uncoupler-stimulated respiration standardized to wet weight, maximal complex IV activity, and citrate synthase activity. Median maximal complex IV activity standardized to wet weight and citrate synthase activity. Data are represented as the median (IQR)

In order to assess mitochondrial function, we performed HRR using PMF of RA participants and controls. These data are shown in Table 3 and Fig. 1. For respirometry data normalized to wet weight, the leak rate with fatty acid (palmitoylcarnitine) [mean difference (95% CI) =  − 3.19 (− 4.50, − 1.89) pmol/(s*mg), *p* < 0.01], maximal rate of uncoupler-stimulated respiration [mean difference (95% CI) =  − 17.27 (− 32.28, − 2.25) pmol/(s*mg), *p* = 0.03] and maximal complex IV activity (mitochondrial content) [mean difference (95% CI) =  − 74.45 (− 108.90, − 40.01) pmol/(s*mg), *p* < 0.01] were all significantly lower among RA participants compared to controls.

**Table 3:** High-resolution respirometry using permeabilized skeletal muscle fibers of RA and control participants

	**Weight standardized**		**Max complex IV standardized**		**CS activity standardized**	
**Variable**^**a**^	**Mean difference** **(95% CI)**	***p*-value**	**Mean difference** **(95% CI)**	***p*-value**	**Mean difference** **(95% CI)**	***p*-value**
Leak rate with fatty acid substrate (PC)	− 3.19 (− 4.50, − 1.89)	< 0.01	0.01 (− 0.004, 0.02)	0.17	− 0.06 (− 0.26, 0.14)	0.59
OXPHOS capacity	− 6.69 (− 22.18, 8.80)	0.41	0.14 (0.02, 0.26)	0.03	0.82 (− 0.91, 2.55)	0.36
Maximal rate of uncoupler-stimulated respiration	− 17.27 (− 32.28, − 2.25)	0.03	0.09 (− 0.04, 0.22)	0.17	− 0.18 (− 1.76, 1.39)	0.82
Max complex IV activity (mitochondrial content)^b^	− 74.45 (− 108.90, − 40.01)	< 0.01			− 3.01 (− 6.79, 0.77)	0.13

^a^Controls are the reference group in all cases. Negative values indicate that the variable is lower in the RA group

^b^As this variable was used to standardize the mitochondrial content, it is only reported for the weight standardized values

**Fig. 2:**
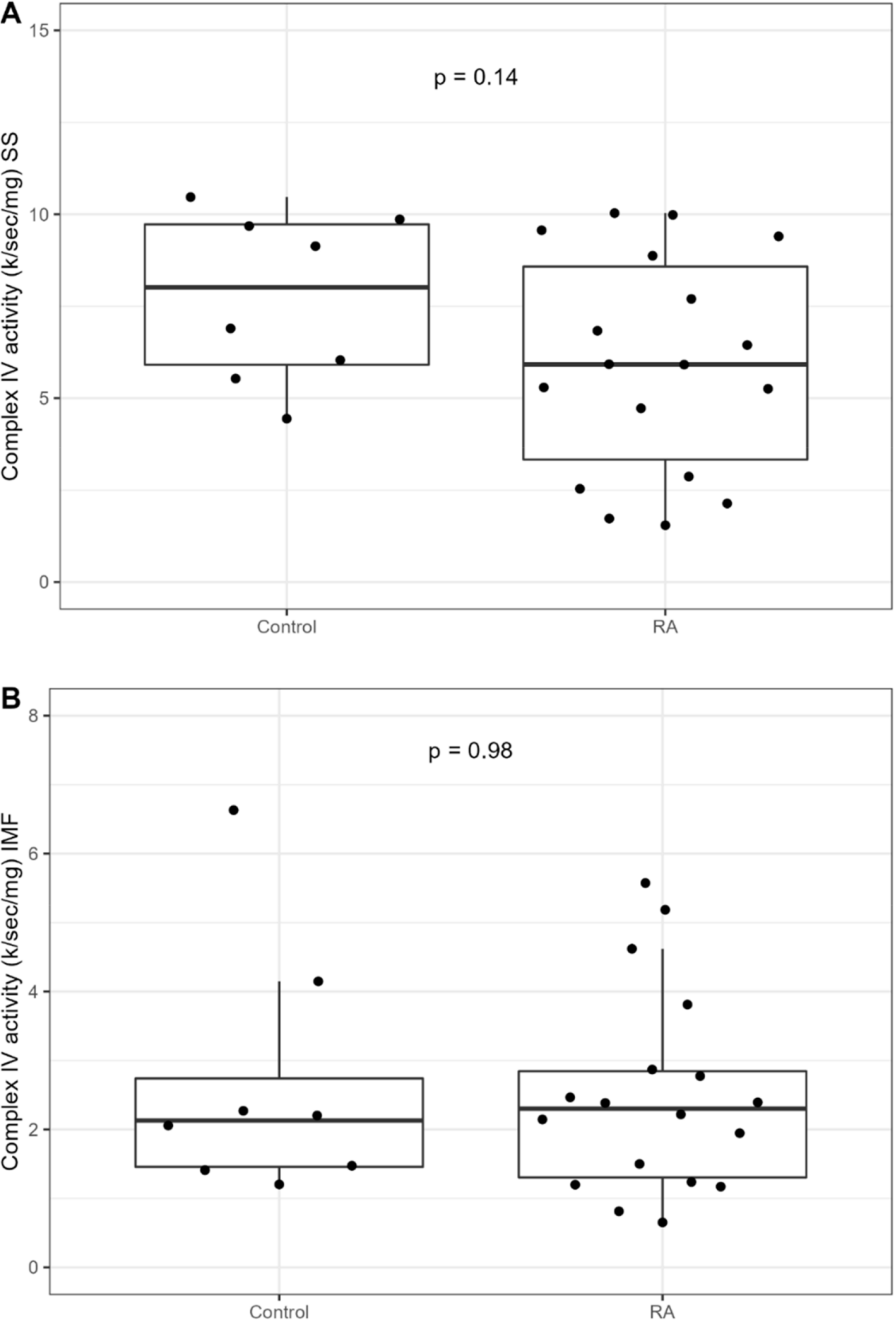
**A**, **B** Complex IV kinetics in the subsarcolemmal (SS) and intermyofibrillary mitochondrial subpopulations (IMF)

For respirometry data normalized to mitochondrial content within the HRR samples using either TMPD/As-stimulated complex IV activity or CS activity, the differences in the leak rate with fatty acid and maximal rate of uncoupler-stimulated respiration were no longer significant between the two groups. Further assessment of mitochondrial function with complex IV enzyme kinetics in isolated subsarcolemmal [5.92 (3.33, 8.58) k/s/mg vs. 8.02 (5.91, 9.73) k/s/mg], *p* = 0.14] and intermyofibrillar mitochondrial subpopulations [2.30 (1.30, 2.84) k/s/mg vs. 2.13 (1.46, 2.74) k/s/mg, *p* = 0.98] revealed no significant differences between RA and controls, as demonstrated in Fig. 2A and B.

Additionally, there were no significant group differences in RCR 3/2 fatty acid (*p* = 0.41), suggesting mitochondrial coupling efficiency was maintained in RA muscle. Taken together, these data indicate that RA patients have lower mitochondrial content compared to individuals without RA, rather than impaired mitochondrial function or electron transport chain enzyme activity. There were also no significant differences in the OxPhos capacity (state 3) normalized to CS activity between the two groups. However, OxPhos capacity (state 3) normalized to TMPD/As-stimulated complex IV activity was higher among RA participants compared to controls [mean difference (95% CI) = 0.02, 0.26, *p* = 0.03]. A possible explanation for the discrepancy between these results could be slight differences in the content of muscle fiber types and mitochondrial subpopulations between muscle aliquots used for the respirometry and CS activity assays. Another explanation could be the differential regulation of OxPhos activity relative to citrate synthase activity. In isolated human lymphocytes, the ratio of complex IV to citrate synthase activity has been shown to be different in obese versus non-obese age-matched controls, where citrate synthase activity was significantly lower and complex IV activity was significantly higher in obese individuals compared to controls [40]. In this study, the ratio of complex IV (TMPD/As) activity relative to citrate synthase activity had a greater range of values in the RA population compared to the controls (data not shown).

### Muscle mitochondrial content

**Fig. 3:**
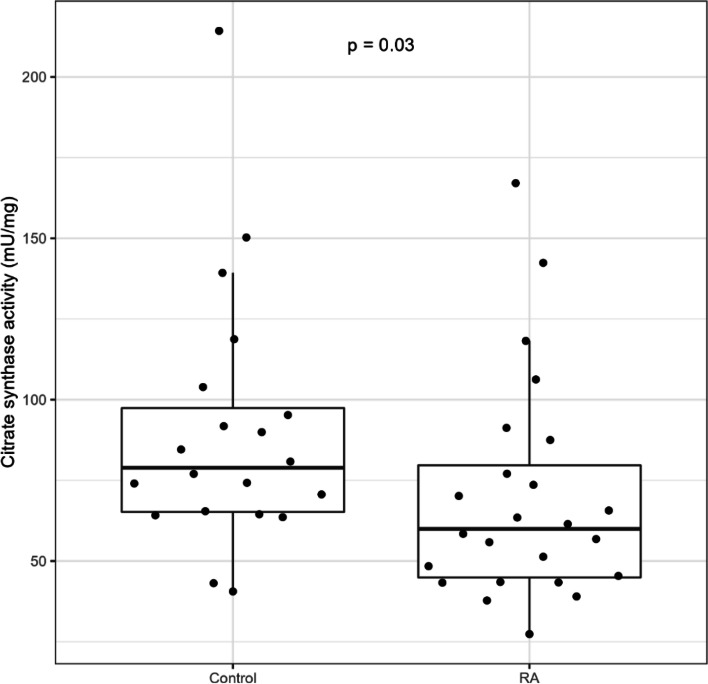
Skeletal muscle citrate synthase (CS) activity in RA versus control. Median CS activity in muscle homogenates of biopsies from vastus lateralis of control and rheumatoid arthritis (RA) participants. Data are represented as the median (IQR)

In order to validate the observation of lower muscle mitochondrial content in RA, we measured CS activity. In line with the respirometry findings, CS activity was significantly lower in RA [60 mU/mg (45, 80)] compared to controls [79 mU/mg (65, 97)], *p* = 0.03, as demonstrated in Fig. 3.

**Fig. 4:**
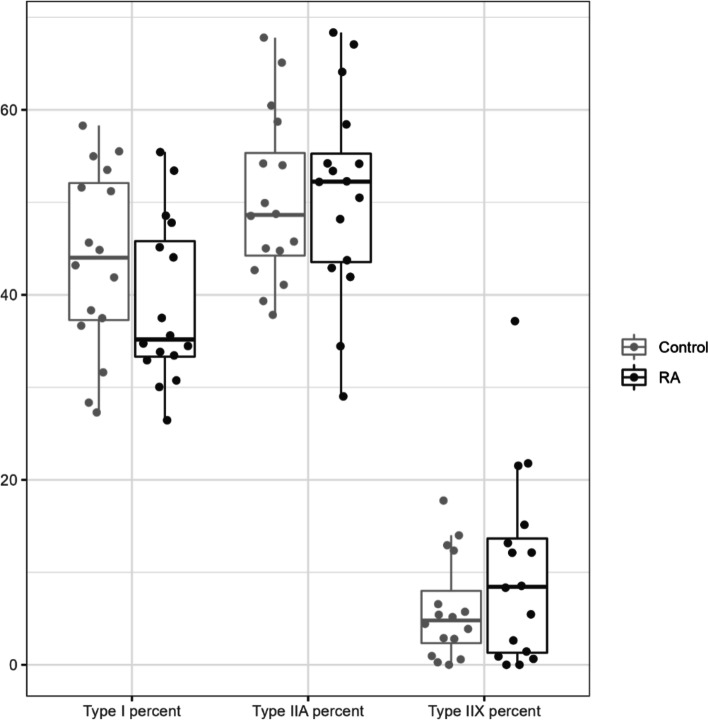
Distribution of myosin heavy chain (MHC) isoforms by immunohistochemical analysis. The median percentage of MHC isoforms I, IIA, and IIX in biopsies from vastus lateralis of RA participants and controls analyzed by immunohistochemistry. Data are represented as median (IQR)

CS activity is fiber type dependent, so we ascertained that lower muscle mitochondrial content was not due to changes in fiber composition. Both immunohistochemical and quantitative electrophoretic analyses of myosin heavy chain isoform distribution confirmed there were no differences between RA participants and controls. Immunohistochemical analysis demonstrated the median proportions of type I, IIA and IIX myofibers were 44% (37,52), 49% (44, 55), and 6% (3,13), respectively, among controls; and 35% (33,46), 52% (44, 55), and 8% (1,14), respectively, among RA participants (Fig. 4).

**Fig. 5:**
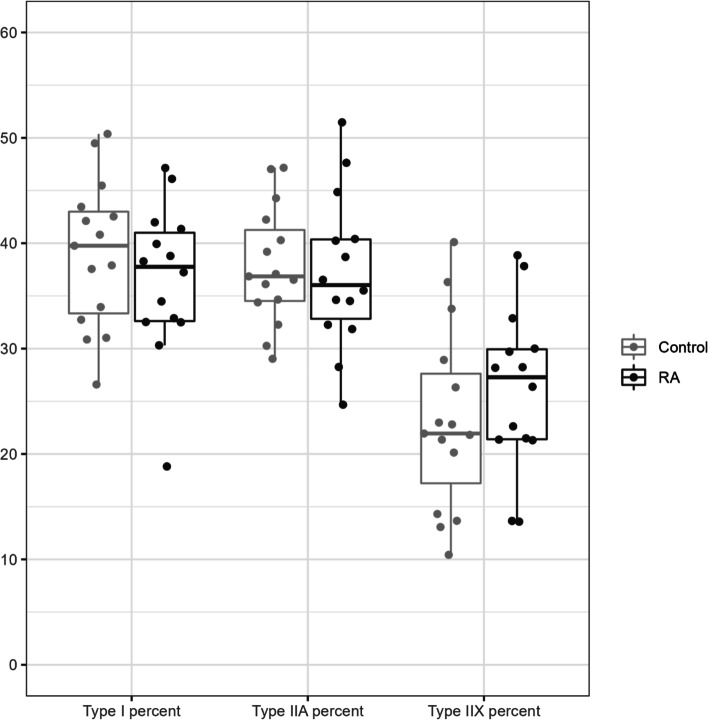
Distribution of myosin heavy chain (MHC) isoforms by electrophoretic analysis

Electrophoretic analysis of MHC isoforms revealed the mean percentage of type I, IIA, and IID/X isoforms were 40% (7.8), 37.6% (5.5), and 22.5% (8.9), respectively, among controls; and 36.6% (7.2), 37.3% (7.3), and 26.1% (7.7), respectively, among RA participants. The results of the electrophoretic analysis are shown in Fig. 5.

**Fig. 6:**
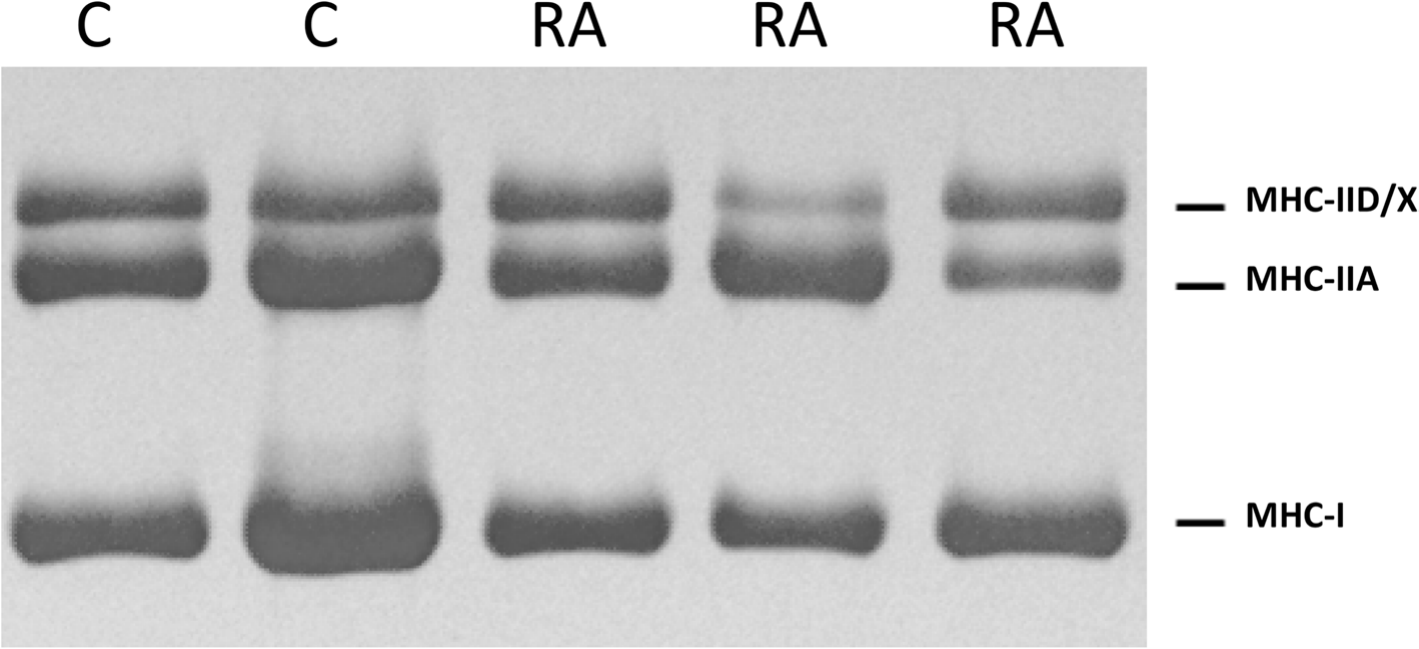
Representative image of myosin heavy chain (MHC) isoforms on a silver-stained gel. The myosin heavy chain region of a silver-stained gel, loaded with homogenates of biopsies from vastus lateralis of control (C) participants and rheumatoid arthritis (RA) participants

Figure 6 depicts a representative image of MHC isoforms on a silver-stained gel.

### Demographic, clinical, and lifestyle-related correlates with mitochondrial content among RA participants

Table 4 demonstrates CS activity was significantly positively correlated with IPAQ total MET-minutes per week (*ρ* = 0.44, *p* = 0.03) and Actigraph-measured time on physical activity (MET rate min/week) (*ρ* = 0.47, *p* = 0.03) among the RA participants. Although statistical significance was not achieved, Actigraph-measured total activity count per day also tended to be positively correlated with CS activity (*ρ* = 0.41, *p* = 0.06). We did not observe significant correlations of CS activity with age, BMI, DAS28-CRP, serum CRP, or the Matsuda index.

**Table 4:** Spearman’s correlations with citrate synthase (CS) activity among RA participants

Variable	Coefficient (*ρ*)	*p*-value
Age	0.12	0.57
BMI	− 0.16	0.44
DAS28-CRP, *n* = 23	− 0.05	0.82
Serum CRP, *n* = 23	− 0.11	0.63
IPAQ, total MET-minutes/week	0.44	0.03
Accelerometer, *n* = 22		
MET rate (min/week)	0.47	0.03
Total activity count/day	0.41	0.06
Average MVPA/day	0.12	0.59
Total step count/day	0.35	0.11
Matsuda index	− 0.05	0.84

## Discussion

Our data confirm previous studies that show a higher level of insulin resistance among RA participants compared to controls [41]. Insulin resistance in RA is multifactorial, with corticosteroid treatment and systemic inflammation likely playing a major role [41]. Skeletal muscle mitochondrial dysfunction has been implicated in insulin resistance [9, 12, 42], but this has not been previously reported in RA. One prevailing hypothesis is that increased fatty acid uptake into muscle and or impaired fatty acid oxidation due to lower mitochondrial content and/or function lead to the accumulation of lipid intermediates that interfere with insulin signaling [8]. Therefore, the goal of this study was to examine muscle OxPhos and mitochondrial content in RA participants in relation to insulin resistance.

The novel finding of this study is that mitochondrial dysfunction did not seem to be a major contributing factor for insulin resistance in RA. Muscle mitochondrial content was lower in RA participants compared to controls, but mitochondrial content was not statistically correlated with insulin resistance among RA participants. Notably, OxPhos normalized to mitochondrial content (“intrinsic” OxPhos) was higher in RA participants compared to controls, whereas previous studies of insulin-resistant offspring of Type 2 diabetics and type 2 diabetics reported opposite results [12, 42]. When taken together, our findings suggest higher “intrinsic” mitochondrial OxPhos seems to be compensating for lower muscle mitochondrial content in RA. A possible explanation for the discrepancy between our results and the earlier findings is that in participants with type 2 diabetes and insulin-resistant offspring of type 2 diabetics, the pathogenesis of insulin resistance is probably linked to heritable defects of mitochondrial metabolism [42]. On the other hand, in RA, an acquired non-heritable impairment of oxidative phosphorylation may become evident only as insulin resistance and inflammation progress and compensatory mechanisms begin to fail. Higher “intrinsic” OxPhos in RA could be also related to mitochondrial lipid overload, as a result of increased lipolysis associated with inflammation and or excess adiposity [43]. Although the effect of elevated lipid availability on muscle mitochondrial capacity in humans is controversial, in rodents, it has been shown to increase muscle fatty acid oxidative capacity, via the coordinated increase in the activity of β-oxidation and tricarboxylic acid (TCA) cycle enzymes, as well as increased expression of respiratory chain subunits [44]. Individuals with RA could be predisposed to lipid overload due to the high prevalence of obesity and other body composition abnormalities, [45] coupled with physical inactivity [46].

Our study, for the first time, demonstrates the association between muscle mitochondrial content and physical level in RA patients, highlighting the potential for exercise interventions to enhance mitochondrial function and improve overall outcome. In the present study, physical activity level was significantly lower among RA participants compared to controls, despite low disease activity levels achieved with DMARD therapy among our participants with RA. Our findings are consistent with several other studies that have reported that a large proportion of RA patients have a sedentary lifestyle and are less physically active compared to controls [46, 47]. Several recent studies have examined risk factors for sedentary behavior in patients with RA, with one study suggesting that fatigue could be both a determinant and a consequence of sedentary behavior [48]. Mitochondrial content is correlated with skeletal muscle oxidative capacity and aerobic performance [49]. Thus, lower muscle mitochondrial content could contribute to fatigue and exercise intolerance in RA patients. Indeed, a recent study demonstrated “alarmingly” low VO2 max levels in 150 RA patients, which was significantly associated with body fat and insulin resistance, even after adjustment for physical activity, RA activity, and severity [50]. This data underscores the important interplay between obesity, physical activity, fatigue, and insulin resistance in patients with RA. High-intensity interval training (HIIT) [51] has been shown to increase mitochondria content and mitochondrial electron transport chain and fatty acid oxidation enzyme activities [52, 53]. Future studies could establish the efficacy, optimal frequency, intensity, time, and type of exercise that would improve mitochondrial function and the health of RA patients.

In terms of study limitations, our sample size is modest, which may result in an inability to detect small or even medium associations. Therefore, with respect to the correlations of CS activity with clinical parameters, the inclusion of a larger series of patients and control participants could have led to stronger results in terms of statistical significance. Additionally, in the high-resolution respirometry study to assess mitochondrial function, we utilized the fatty acid substrate palmitoylcarnitine, which is not dependent on fatty acid transporters. Therefore, we did not examine the carnitine shuttle system for abnormalities that could lead to a reduction in mitochondrial function. However, our study has many strengths, including the characterization of mitochondrial function by high-resolution respirometry, as well as the assessment of complex IV activity in the two distinct mitochondrial subpopulations. A better understanding of muscle mitochondria in RA sets the stage for measuring the efficacy of therapeutic interventions, such as exercise and diet.

## Conclusions

Mitochondrial dysfunction did not seem to be a major contributing factor for insulin resistance in RA. Muscle mitochondrial content was lower in RA participants compared to controls, but mitochondrial content was not statistically correlated with insulin resistance among RA participants. This study demonstrates the association between muscle mitochondrial content and physical level in RA patients, highlighting the potential for exercise interventions to enhance mitochondrial function and improve overall outcome.

## Supplementary Information

**Additional file 1: Table 1S.** Characteristics of participant subgroup with mitochondrial respirometry data.

## Data Availability

The datasets used and/or analyzed during the current study are available from the corresponding author on reasonable request.

## Abbreviations

ACPA: Anti-citrullinated protein antibodies
Anti-CCP: Anti-cyclic citrullinated peptide
BMI: Body mass index
CS: Citrate synthase
DAS28-CRP: Disease activity score 28-joint count C-reactive protein
DMARD: Disease-modifying antirheumatic drugs
DXA: Dual-energy X-ray absorptiometry
ETC: Electron transport chain
FCCP: 2-[2-[4-(Trifluoromethoxy) phenyl] hydrazinylidene]-propanedinitrile
FFMI: Fat-free mass index
HRR: High-resolution respirometry
hsCRP: High-sensitivity C-reactive protein
IMF: Intermyofibrillary
IQR: Interquartile range
MET-min/week: Metabolic equivalent minutes per week
MHC: Myosin heavy chain
MVPA/day: Moderate-to-vigorous physical activity/day
OGTT: Oral glucose tolerance test
OxPhos: Oxidative phosphorylation
PMF: Permeabilized muscle fibers
RA: Rheumatoid arthritis
RF: Rheumatoid factor
SS: Subsarcolemmal
TMPD: Tetramethyl-p-phenylenediamine
