# Supplementary material for: Association between skeletal muscle mitochondrial dysfunction and insulin resistance in patients with rheumatoid arthritis: a case–control study
Source: Arthritis Res Ther. 2023 May 20;25:85. doi: 10.1186/s13075-023-03065-z (PMC10199606; doi:10.1186/s13075-023-03065-z)
Supplement: Supplementary file 1 — Additional file 1: Table 1S. Characteristics of participant subgroup with mitochondrial respirometry data. [file 13075_2023_3065_MOESM1_ESM.docx]

**Table 1S.** Characteristics of participant subgroup with mitochondrial respirometry data.

| **Characteristic** | **Control (n=9)** | **RA (n=18)** | **p-value** |
| --- | --- | --- | --- |
| Age, years  Female sex (%)  Race (%)  White  Black or African American  Smoking status (%)  Never smoked  Past smoker  Current smoker  Co-morbidities  Dyslipidemia (%)  Systemic hypertension (%)  COPD/ emphysema (%)  Anthropometrics  BMI, Kg/m2  Waist-to-hip ratio  Fat free mass index (Kg/m^2^)  Fat mass index (Kg/ m^2^)  Physical activity measures  IPAQ, total MET-min/ wk  IPAQ Moderate intensity MET-min/ wk  IPAQ Vigorous intensity MET-min/ wk  Accelerometer (n=9, 17)  MET Rate (kcals hourly equivalent)  Total activity count/ day (in millions)  MVPA/day  Step counts/ day  Serum C-reactive protein mg/L (n=9, 15)  Matsuda Index | 51 (44, 60)  9 (100)  7 (78)  2 (22)  6 (67)  2 (22)  1 (11)  0 (0)  0 (0)  0 (0)  26.4 (24.6, 30.8)  0.83 (0.81, 0.85)  15.3 (14.7, 16.2)  11.8 (8.7, 11.9)  2127 (1,920, 4,803)  720 (27, 2,280)  0 (0, 1,920)  1.10 (1.06, 1.13)  0.87 (0.78, 1.25)  101 (76, 125)  8725 (7,037, 9,645)  1.49 (1.19, 2.02)  6.04 (5.83, 7.75) | 55 (50, 59)  18 (100)  16 (89)  2 (11)  9 (50)  6 (33)  3 (17)  6 (33)  5 (28)  0 (0)  28.6 (24.9, 31.9)  0.89 (0.84, 0.91)  16.2 (15.3, 17.1)  12.1 (10.0, 14.6)  1208 (785, 2,253)  810 (346, 1,635)  0 (0, 0)  1.05 (1.02, 1.07)  0.93 (0.72, 1.07)  104 (72, 115)  5604 (4,133, 9,136)  0.93 (0.53, 2.11)  3.95 (2.33, 5.64) | 0.50  >0.99  0.58  0.86  0.07  0.14  >0.99  0.46  0.06  0.18  0.63  0.17  0.92  0.05  0.03  0.71  0.03  0.06  0.19  0.02 |
